# Influence of a caring, task-involving motivational climate on army cadets’ self-efficacy, arousal, attentional focus, and marksmanship performance

**DOI:** 10.3389/fpsyg.2026.1819408

**Published:** 2026-07-15

**Authors:** E. Whitney G. Moore, Taylor Kinney, Christine M. Habeeb, Nicholas P. Murray

**Affiliations:** Department of Kinesiology, College of Health and Human Performance, East Carolina University, Greenville, NC, United States

**Keywords:** achievement goal perspective theory, ego-involving, eye tracking, heart rate, heart rate variability, respiratory rate, self-confidence, shooting simulation

## Abstract

**Introduction:**

Initial qualitative research has supported the extension of motivational climates to the military context. Thus, there is a need for transdisciplinary research to examine the motivational climate that fosters psychophysiological responses in military personnel while conducting representative tasks.

**Methods:**

A total of 64 Reserve Officer Training Corps (ROTC) cadets completed a baseline marksmanship task (Time 1) and repeated the same task 1 month later (Time 2) in either a caring, task-involving (CTI) or ego-involving (EI) motivational climate condition created by a senior ROTC cadet leader. At both time points, cadets’ heart rate variability (i.e., arousal state), gaze fixation (i.e., attentional focus), shooting task self-efficacy, and performance (i.e., hitting the target) were recorded.

**Results:**

Analysis of Time 2 climate survey responses confirmed that the ROTC cadet leaders fostered significantly different climate conditions. Analysis of covariances (ANCOVAs), controlling for baseline values, revealed meaningful differences across conditions (i.e., variance explained by condition) and often significant differences. At Time 2, CTI condition cadets had higher self-efficacy, arousal, gaze fixation time, and averaged three more target hits (out of 45) compared to the EI condition.

**Discussion:**

Overall, those in the CTI condition were physiologically more aroused, with greater attentional focus, which positively influenced their self-efficacy and performance.

## 1 Introduction

The psychosocial climates of achievement goal perspective theory (AGPT) and the caring climate framework have shown consistent psychosocial responses across academic, sport, and exercise contexts (Duda and Nicholls, 1992; Fry and Moore, 2019; Gu and Cheng, 2026; Harwood et al., 2015; Nicholls, 1989). Researchers have demonstrated that these climates promote different psychological interpretations of demands and resources among participants, leading to different stress responses, as evidenced by both self-report and physiological markers (Hogue et al., 2013; Hogue, 2024; Hogue et al., 2025b). These findings align with Nicholls’ original proposition to identify and understand a motivational climate (i.e., task-involving) that could optimize participants’ motivational responses and physiological state for learning and performing. Recently, researchers have provided evidence supporting the application of AGPT motivational climates to the military context (Fischer, 2015; Le Foll et al., 2019; Nerstad et al., 2020). Thus, the purpose of this study was to understand better how experiencing either a caring, task-involving (CTI) or an ego-involving EI motivational climate influences individuals’ psychophysiological responses while performing a military-relevant task—rifle shooting.

Nicholls (1989) goal was to identify the components of the climate in achievement contexts that did and did not result in optimal motivational responses. He described two climates, namely, task-involving climate and ego-involving climate. The task-involving climate occurs when leaders emphasize effort, mastery, cooperative learning, and the treatment of mistakes as learning opportunities. The ego-involving climate occurs when leaders reward ability over effort, compare performance to others and norms, and punish mistakes. Building on Nicholls’ study, Newton et al. (2007) defined a caring climate as one in which participants feel safe, welcomed, respected, and valued for who they are as people, not just for their performance or capabilities. Since then, researchers have examined the caring climate alongside the task-involving climate, as they often co-occur in practice (Fry and Moore, 2019). For example, leaders use a person’s name (caring) while providing constructive feedback (task-involving). The caring and task-involving climates are positively correlated, and both are negatively correlated with the ego-involving climate.

Researchers have consistently found evidence supporting that when people are in a caring, task-involving (CTI) climate, they give more effort, persist longer, enjoy the task, are more self-confident, select challenges appropriate for building their mastery, and have lower anxiety (Albert et al., 2021; Fry and Moore, 2019; Hogue et al., 2025b; O’Rourke et al., 2014; Pedditzi, 2014; Smith et al., 2007; Wood and Bandura, 1989; Zourbanos et al., 2016). Whereas individuals experiencing an ego-involving (EI) climate report more tension/pressure, anxiety, shame, burnout, and less confidence/self-efficacy (Fry and Moore, 2019; Harwood et al., 2015; Hogue et al., 2025b; O’Rourke et al., 2014; Pedditzi, 2014; Smith et al., 2007; Wood and Bandura, 1989; Zourbanos et al., 2016). These relationships support the experience of CTI climates promoting more adaptive responses among people than EI climate experiences across academic and sport contexts.

To date, three studies have explored the application of AGPT in the military context (Fischer, 2015; Le Foll et al., 2019; Nerstad et al., 2020). Primarily, these studies have been conducted with samples of Reserve Officer Training Corps (ROTC) cadets. Graduates of ROTC programs within universities produce more commissioned officers than any other individual source (Kapp, 2022). ROTC is a demanding program that prepares full-time university students for an Officer position in active duty by developing leadership skills and military performance (e.g., physical fitness, marksmanship). The major guidance for ROTC programing defines leadership as an “activity of influencing people by providing purpose, direction, and motivation to accomplish the mission and improve the organization” (Department of the Army, 2025, p. xi). ROTC cadets are trained in the attributes and competencies they should develop as leaders, such as transformational leadership. Although motivational climates are not currently addressed in this programing, researchers have shown that transformational leadership behaviors promote a TI climate (Álvarez et al., 2019; Newland et al., 2020). Initial qualitative research on the experiences of ROTC cadets in the US suggested they experienced a mixed motivational climate during their physical training, including both ego-involving and task-involving characteristics (Fischer, 2015). The ego-involving characteristics included normative comparisons with Ability Group Runs and Physical Fitness Testing, with passing norms required to maintain scholarships. The task-involving characteristics included emphasizing individual self-improvement through physical challenges, faculty and senior cadets leading by example with their high effort during physical training, and providing social support. However, based on both qualitative interviews and observations, Fischer (2015) recommended increasing the task-involving climate characteristics to optimize cadet learning and performance and to develop cadets as leaders. The importance of recommending the promotion of TI climate during cadets’ physical training was further supported by a cross-sectional finding by Frederick et al. (2021) that the cadets’ intrinsic motivation and enjoyment of physical training were negatively associated with their years in ROTC.

The other two studies of AGPT with military personnel have provided support for the theoretical expectation that relationships established in other achievement contexts continue to be observed in military settings. Specifically, Nerstad et al. (2020) provided longitudinal support for the relationship between cadets’ perceptions of the motivational climate and their definitions of success. As seen previously in classroom, sport, and physical education (PE) settings, TI climate perceptions positively predicted future task goal orientation levels (i.e., cadets held a self-referenced definition of success). In addition, EI climate perceptions positively predicted future levels of ego goal orientation (i.e., cadets held an other-referenced definition of success). Furthermore, Le Foll et al. (2019) found in a sample of French soldiers that their TI climate perceptions significantly and positively predicted a large portion of the variance in their reported unit cohesion. These soldiers also reported significantly higher TI than EI climate values. To date, studies of AGPT with military samples have supported consistency in the theoretical constructs and their relationships when applied to this newest context. Thus far, no study to date has included physiological measures or performance associated with the military.

Experimental studies controlling the climate conditions experienced by young adult participants have shown that CTI and EI climates promote distinct psychoneuroendocrine responses (e.g., cortisol, testosterone, alpha-amylase) and behaviors (Hogue et al., 2013; Hogue, 2024; Hogue et al., 2025a; Hogue et al., 2025b; Solmon, 1996). When learning a juggling task, Solmon (1996) demonstrated that participants in a TI climate persisted, tried appropriate challenges to build their skill, and enjoyed the activity, unlike those in the EI climate. Following up with additional studies on juggling tasks, Hogue et al. (2013) demonstrated that young adults’ cortisol levels increased significantly in the EI climate compared to the CTI climate. EI participants’ self-reported shame, anxiety, stress, and self-consciousness were all significantly higher compared to the CTI participants. Recently, Hogue et al. (2025b) extended CTI and EI experimental condition research to include another low-intensity task (basketball free throws) to not prompt physiological causes of stress, so cortisol changes would only be due to psychological stress from the climate condition. Again, compared to young adults in the CTI climate, EI climate participants had significantly higher cortisol, testosterone, and alpha-amylase levels, indicating an increased stress response among those experiencing the EI climate compared to the CTI climate (Hogue et al., 2025b). Furthermore, the CTI participants improved their basketball shooting biomechanics more compared to the EI participants (Hogue et al., 2025a). These studies with manipulated climate conditions provide evidence of suboptimal responses among young adults when learning a new skill or improving an existing one, in an EI climate compared to a CTI climate. These suboptimal responses included significant stress psychoneuroendorcrine responses, as well as reduced performance in the skill being developed.

A military-specific, low-intensity task similar to juggling and basketball free-throw shooting is rifle target shooting in prone, kneeling, and standing positions. Rifle shooting is a technical task. Researchers have shown rifle shooting performance by both soldiers and elite athletes is connected to their stress and anxiety responses as measured by their heart and respiratory rates, as well as their ability to maintain their eyes’ focus on the target (European Olympic Committee, 2019; Ihalainen et al., 2016; Saxena, 2022). Specifically, Ihalainen et al. (2016) found that 80% of elite shooters’ performance could be explained by eye-tracking factors. Shooters’ self-efficacy level for the shooting task has also been related to their shooting performance (Saxena, 2022). Quantitatively, Saxena (2022) found that shooters’ season ranking was more strongly correlated with their self-efficacy than flow, grit, or passion. Thus, successful shooting is achieved through regulation of emotional and physiological states (heart/respiratory rates), time on target, attentional focus, and self-efficacy. In qualitative interviews, elite shooters highlighted those instructing them on their shooting performance (e.g., coaches and instructors) as a source for anxiety (Saxena, 2022). Therefore, rifle shooting performance has been linked to shooters’ arousal state (i.e., heart rate variability), attentional focus (i.e., eye fixation duration measured with tracking glasses), self-efficacy, and leader-fostered motivational climate.

However, no study to date has examined how the motivational climate influences all of the above variables and, in turn, their shooting performance. The purpose of this study was to examine the effect of experiencing a leader-foster CTI climate compared to an EI climate condition on ROTC cadets’ arousal state (i.e., heart rate variability), attentional focus (i.e., eye fixation duration measured by tracking glasses), self-efficacy, and performance (i.e., shots hitting the target). It was hypothesized that, controlling for baseline, those in the CTI climate, compared to the EI climate, would perform better on the shooting task while experiencing a more optimal motivational state based on their arousal level, attentional focus, and self-efficacy.

## 2 Materials and methods

This research was part of a larger funded study (Moore et al., 2026); the larger grant procedure details are available on the project registration osf site https://osf.io/wmts4/overview.

### 2.1 Participants

Sixty-four ROTC cadets completed an air rifle performance assessment across two time points. Cadets’ average previous qualifier rating was Marksman out of the possible ratings of Marksman (11 in CTI; 11 in EI), Sharpshooter (4 in CTI; 5 in EI), and Expert (2 in CTI, 2 in EI). A total of 31 cadets (45% women) were in the CTI condition, and 33 cadets (27% women; 24% Hispanic) were in the EI condition at Time 2. Cadets in the CTI condition identified their race as multiracial (6.5%), white (71%), and Black (19%). Cadets in the EI condition identified their race as multiracial (15%), white (67%), Black (24%), American Indian/Alaska Native (9%), Asian/Asian American (18%), and Native Hawaiian/Pacific Islander (3%). Regarding training experience, 61% of those in the CTI and 64% in the EI condition had Army rifle training. Power analyses conducted in GPower based on Hogue et al. (2013) effect size (eta-squared [*η*^2^]) of 0.35, with alpha of 0.05, and power of 0.80 supported needing a total sample size of 40. Thus, the study sample size of 64 (31 CTI, 33 EI) was sufficiently powered to detect such an effect size.

### 2.2 Procedure

After receiving IRB approval, ROTC cadets were invited to participate at Time 1 (no feedback) and again at Time 2 (CTI or EI climate condition) a month later. Cadets came in groups of three, based on the available measurement equipment and shooting lanes. At Time 1, cadets provided informed, written consent and completed a demographic survey before meeting the research team members responsible for data collection in their assigned shooting lane. One research team member set up the 3-lead EKG and respiratory chest strap, while another team member set up the cadet with their eye-tracking glasses and completed the calibration step for the glasses. Then, all three cadets sat down with their backs against their shooting stand to complete the 60-s calibration of the EKG and respiratory chest strap. After calibration was complete, the shooting procedure was explained, and cadets were shown the format of the self-efficacy item. Next, the cadets completed the zeroing process with their air rifles using the simulator program. After successful zeroing and calibration by all three cadets, they moved into the prone position, were asked about their self-efficacy for shooting in the prone position, and then the simulator started. They took five shots in the current position at the 50 m simulated distance. Then, the cadets’ results were projected by the simulation program as the number of successful target hits out of the five attempts. The research team member responsible for recording the cadet’s self-efficacy was responsible for taking and uploading a picture of the cadet’s performance projected on the simulator screen. This process was repeated for the 5 shots at 100 m and the 5 shots at 150 m. Then, all cadets transitioned to the kneeling position and repeated the steps for efficacy reporting, shooting, and performance recording. Finally, cadets transitioned to the standing position and repeated the efficacy reporting, shooting, and performance recording. Altogether, the marksmanship task itself took no more than 15 min to complete.

At Time 2, the cadets arrived for their follow-up session and were informed that while they repeated the marksmanship task, they would be receiving feedback from a randomly assigned senior cadet leader; the senior cadet leader was going to provide feedback in the manner assigned by the research team (i.e., CTI or EI). The CTI and EI conditions were created by the senior cadets during the marksmanship task activity. This structure mimicked the real-world format structure for rifle marksmanship practice sessions, during which instructors provide feedback between brief shot sets (so shooters can hear it) (Department of the Army, 2016). After the marksmanship activity was completed, the cadets went to another room to complete a post-survey, including the manipulation-check measures.

### 2.3 Experimental manipulation

Two of the senior researchers conducted a 90-min leadership workshop on CTI and EI climates for the senior cadet class before Time point 2. The senior researchers asked the cadets to write and then share about the characteristics of their favorite and least favorite sport/PE leaders, as well as their responses to those two experiences. These components (recorded on the class whiteboard) aligned with the CTI and EI climates, which set up the senior researchers to connect the senior cadets’ descriptions with the different climate definitions and relationships supported by research in sport psychology. Then, the effects of CTI and EI climates were linked to factors influencing rifle marksmanship performance. Next, the senior cadets worked in groups to write down examples of CTI and EI feedback related to the marksmanship cues they were trained in (e.g., breathing, shooting window, and trigger pull). After sharing these with the class, the cadets completed a final activity to practice giving feedback to promote the CTI and EI climates. For this activity, cadets worked in groups of three; two would pretend to shoot while making common errors, while the third cadet practiced being the leader responsible for creating a randomly assigned climate (by picking a card from the researchers). After 1 min, the cadets debriefed on the errors and climate they were each trying to demonstrate, and recorded how successful they were at creating their assigned climate so their “follower” cadets could identify it. Upon completing this activity, with each cadet leading for three rounds, the cadets debriefed as a group about their experiences—what worked, what was challenging, and how to improve—in fostering CTI and EI climates.

After attending the leadership workshop, a dozen senior cadets volunteered and consented to participate as leaders during the Time 2 phase. They each completed a day promoting EI climate and a day promoting CTI climate; the order of these days was randomly assigned. When the senior cadets arrived, they received a half-page document (https://osf.io/5bqaz/overview?view_only=1d92eaaaa0f24ec698060436a4df2fc8) outlining their climate assignment for the day and examples of feedback they had developed during their class activities to help prompt them in their climate development during sessions with the younger cadets who were completing the marksmanship task. For example, the CTI climate was fostered by leaders providing individualized feedback to correct form and positive reinforcement, whereas the EI climate was fostered by leaders fostering rivalry by comparing cadets’ marksmanship performance and pointing out form errors (without correction). Senior cadets primarily provided feedback to the cadets shooting between sets of five shots to ensure they heard the feedback, and if necessary, the senior cadet could demonstrate a change in form or try implementing form changes before the next set of five shots. One of the senior researchers who led the leadership workshop supported the senior cadets as they created their assigned climates each day during the Time 2 phase of the study. In the future, for clarity, these cadets are referred to as senior cadets or cadet leaders to distinguish them from the cadets engaged in the shooting task.

### 2.4 Manipulation check

To check the cadets’ perceptions of the motivational climate fostered by their cadet leader during the Time 2 session, all shooting cadets completed the Perceived Motivational Climate in Exercise Questionnaire-Abbreviated (PMCEQ-A; Moore et al., 2015) and the Caring Climate Scale (CCS; Newton et al., 2007). The 12-item PMCEQ-A measures individuals’ perceptions of the TI and EI climates with 6 items for each construct on a response scale from (1) Strongly Disagree to (5) Strongly Agree. The 13-item CCS measures individuals’ perception of the caring climate construct on a response scale from (1) Strongly Disagree to (5) Strongly Agree. The average score is calculated for each variable, with higher scores indicating greater endorsement of that variable.

### 2.5 Response measures

#### 2.5.1 Arousal assessment (heart rate variability)

The sympathetic-to-vagal response ratio was derived from power spectrum density (PSD) estimates and represents a normalized power ratio. The PSD indicates the power of a signal as a function of frequency, from which a ratio of sympathetic and parasympathetic activation can be determined. The sympathetic-to-vagal response ratio is the ratio of power between the two bands controlling sympathetic and parasympathetic input to the heart. Heart and respiratory rates were collected using Biopac MP 150 (Goleta, CA, USA), a three-lead EKG with a respiration chest strap to measure sympathetic activity.

#### 2.5.2 Attentional focus measurement

Average fixation duration was calculated for each cadet across marksmanship position and distance combinations. This represents the average continuous time cadets’ eyes were held stable (i.e., within 3° of visual angle) on the target, with the minimum duration to qualify for a fixation as 100 milliseconds (Salvucci and Goldberg, 2000). Fixations were captured through three different eye-trackers (Tobii Pro 3 Glasses, Tobii AB, Daeneryd, Sweden; Neon, Pupil Labs GmbH, Berlin, Germany; Eye-Tracking Glasses; SensoMotoric Instruments, Teltow, Germany) that recorded gaze at 100, 200, and 120 Hz, respectively, using dark pupil and corneal reflection technology. Longer average fixation duration has been positively associated with skilled task performance (Vickers, 2007).

#### 2.5.3 Self-efficacy measurement

Following Bandura’s (2006) recommendations, we designed task-specific items assessing the cadets’ position-specific shooting self-efficacy using a single-item measure of self-efficacy (Habeeb et al., 2019). Cadets rated their self-efficacy from 0 (*not at all confident*) to 10 (*completely confident*). After cadets were in position for shooting (i.e., prone, kneeling, or standing) and before the simulator program started, research team members asked the cadets to rate their shooting efficacy in that position, which the research team member recorded in a Qualtrics form.

#### 2.5.4 Performance measurement

Performance was measured as the number of shots that hit the target in prone, kneeling, and standing positions at 50-, 100-, and 150-m distances. Five shots were attempted at each position and distance combination. The sum of target hits across 45 attempts was also calculated. This was conducted using the ROTC program’s air rifle simulator (Baity, 2014).

### 2.6 Statistical analysis plan

The data collected was 96.90% complete. The primary source of missingness was technical errors with the eye-tracking glasses. For example, the two most common patterns of missingness (9% each) reflected data missing due to technical errors with the eyeglasses. Overall, the data met the normality criteria based on skewness (|3|) and kurtosis (|7|) values (Tabachnick and Fidell, 2013). The exceptions to this were primarily the sympathetic-to-vagal ratio and fixation measures at some time points. However, review of these values by a senior research team expert deemed them all within acceptable and realistic ranges.

First, a manipulation check was conducted to confirm that the participants perceived the CTI and EI conditions the leaders were assigned to foster. This check was conducted with an analysis of variance (ANOVA) on the cadets’ caring, task-involving, and ego-involving scores across the two conditions; significant Levene’s Test of homogeneity of variances informed using Welch *F*-tests for significance decisions (Salkind and Frey, 2022). Second, ANCOVAs were conducted to control for Time 1 values while testing for significant differences between climate conditions across the four outcomes: arousal level (heart rate variability), attentional focus (eye-tracking fixation duration), self-efficacy, and performance (hits per position and total). Third, eta-squared (*η*^2^) was calculated to determine the variance of the outcome accounted for by condition, and values were interpreted as small (0.01), medium (0.06), and large (0.14).

## 3 Results

### 3.1 Manipulation check

**Figure 1:**
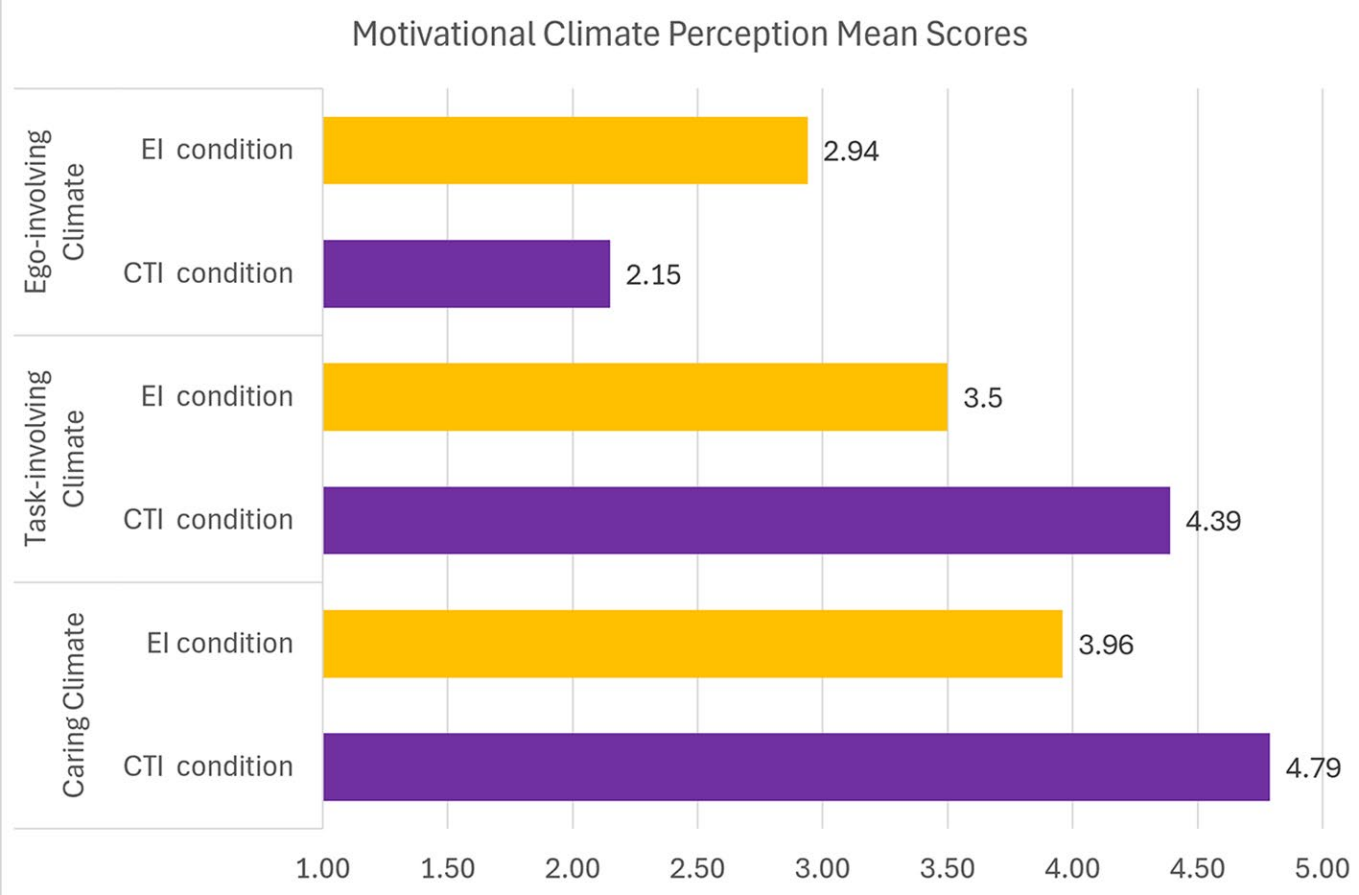
Manipulation check of the motivational climate means by condition.

To check whether the cadets perceived significantly different climates that aligned with the leaders’ assignments, the three climate components—caring, task-involving, and ego-involving—were each tested for significant differences using a one-way ANOVA. The Levene’s test of homogeneity was significant for the caring climate (*F*(1,62) = 35.912, *p* < 0.001) and TI climate (*F*(1,65) = 12.324, *p* < 0.001), but not significant for the EI climate (*F*(1,62) = 1.099, *p* = 0.299). The Welch *F*-tests supported that the two conditions were perceived significantly different by the cadets (see Figure 1). Specifically, the caring climate score was significantly (*F*(1,36.785) = 17.020, *p* < 0.001, ω^2^ = 20.6%) higher for the CTI (*M* = 4.79) than the EI (*M* = 3.96) condition; the task-involving climate score was significantly (*F*(1, 48.426) = 21.413, *p* < 0.001, ω^2^ = 25.0%) higher for the CTI (*M* = 4.39) than the EI (*M* = 3.50) condition; the ego-involving climate score was significantly (*F*(1, 61.984) = 16.496, *p* < 0.001, ω^2^ = 19.4%) lower for the CTI (*M* = 2.15) than the EI (*M* = 2.94) condition. The cadet leaders successfully fostered a high CTI condition while fostering a more mixed climate for the EI condition (with some caring climate and slight task- and ego-involving characteristics). The EI condition composition aligns with previously observed physical training climate among ROTC cadets (Fischer, 2015). In post-survey short answers about the EI condition, cadets noted that their mistakes were identified by the leaders, compared with receiving no feedback during Time 1. Given that this experimental design was conducted in an applied setting, any attributed differences in outcomes are due to a distinct CTI climate condition compared to the realistic EI condition.

### 3.2 Arousal response

**Figure 2:**
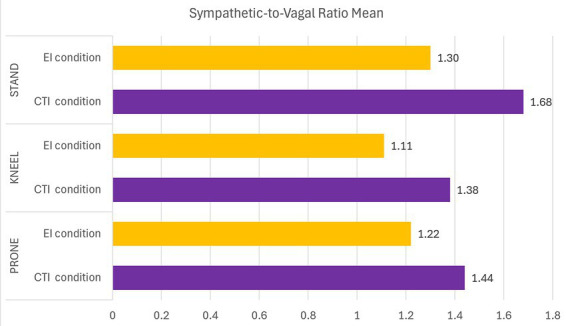
Arousal states by motivational climate condition.

Box’s test of equality of covariances was significant (*p* = 0.012), while Levene’s test of homogeneity of variances was not significant for prone (*p* = 0.320) and kneeling (*p* = 0.753), but was significant for the standing (*p* = 0.032) position. Although, the sympathetic-to-vagal ratios did not significantly differ by condition, a small proportion of variance was accounted for by condition: prone (*F*(1, 55) = 0.763, *p* = 0.386, *η*^2^ = 1.4%), kneeling (*F*(1, 55) = 1.461, *p* = 0.232, *η*^2^ = 2.6%), and standing (*F*(1, 55) = 1.314, *p* = 0.257, *η*^2^ = 2.3%). In each position, the CTI condition participants’ sympathetic-to-vagal ratio was in a more optimal arousal state (i.e., higher sympathetic state) than the EI condition participants (see Figure 2).

### 3.3 Attentional focus response

#### 3.3.1 Prone position

**Figure 3:**
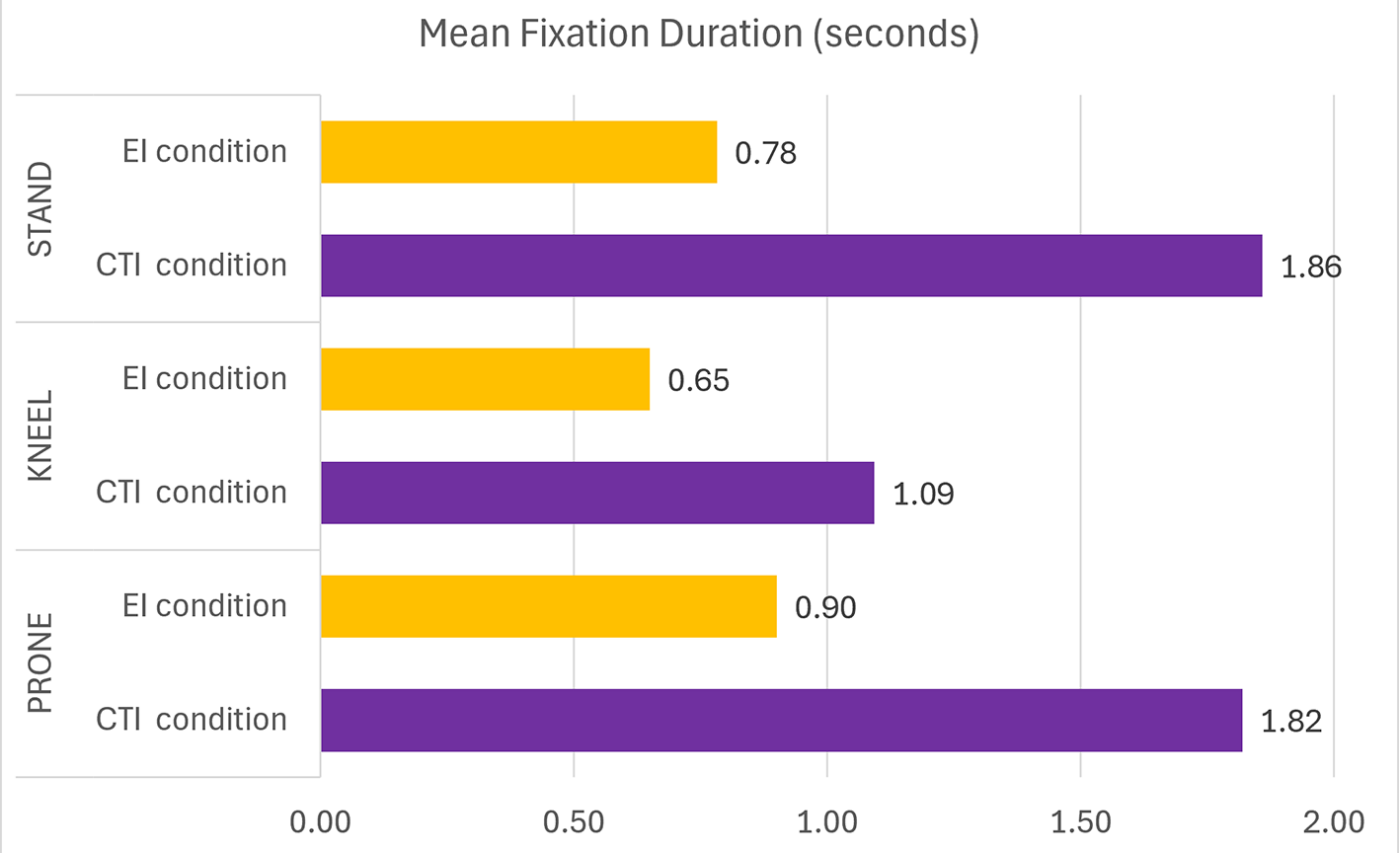
Average fixation duration across target distances for each position by motivational climate condition.

Box’s test of equality of covariances was significant (*p* < 0.001), while Levene’s test of homogeneity of variances was not significant for 50 m (*p* = 0.078), 100 m (*p* = 0.131), and 150 m (*p* = 0.281). While the fixation durations did not significantly differ by condition, a small to moderate proportion of variance was accounted for by condition: 50 m (*F*(1, 38) = 3.186, *p* = 0.082, *η*^2^ = 7.7%), 100 m (*F*(1, 38) = 1.728, *p* = 0.197, *η*^2^ = 4.3%), and 150 m (*F*(1, 38) = 1.045, *p* = 0.313, *η*^2^ = 2.7%). At each distance, the CTI condition participants’ fixation duration was longer, representing greater attentional focus than the EI condition participants (see Figure 3). Practically, the fixation duration was nearly twice as long for the 50 m (*M*_CTI_ = 1.81 s, *M*_EI_ = 0.74 s), 100 m (*M*_CTI_ = 1.76 s, *M*_EI_ = 0.93 s), and 150 m (*M*_CTI_ = 1.89 s, *M*_EI_ = 1.04 s) among the CTI compared to EI participants.

#### 3.3.2 Kneeling position

Box’s test of equality of covariances was significant (*p* < 0.001), while Levene’s test of homogeneity of variances was not significant for 50 m (*p* = 0.078) and 150 m (*p* = 0.772), but was significant for the 100 m (*p* = 0.005) distance. A small to moderate proportion of variance was accounted for by condition, although fixation durations did not significantly differ by condition: 50 m (*F*(1, 37) = 0.317, *p* = 0.577, *η*^2^ = 1.0%), 100 m (*F*(1, 37) = 3.128, *p* = 0.085, *η*^2^ = 7.8%), and 150 m (*F*(1, 37) = 0.263, *p* = 0.611, *η*^2^ = 1%). At each distance, the CTI condition participants’ fixation duration was longer, indicating greater attentional focus than that of the EI condition participants (see Figure 3). Practically, the fixation duration was nearly triple as long for the 100 m (*M*_CTI_ = 1.66 s, *M*_EI_ = 0.59 s) among the CTI compared to EI participants; the differences were not as large for the 50 m (*M*_CTI_ = 0.730 s, *M*_EI_ = 0.62 s), and 150 m (*M*_CTI_ = 0.89 s, *M*_EI_ = 0.74 s).

#### 3.3.3 Standing position

Box’s test of equality of covariances was significant (*p* < 0.001), while Levene’s test of homogeneity of variances was not significant for 50 m (*p* = 0.161) and 150 m (*p* = 0.141), but was significant for 100 m (*p* < 0.001). The fixation durations significantly differed by condition, and a large proportion of variance was accounted for by condition for the 100 m (*F*(1, 25) = 7.220, *p* = 0.013, *η*^2^ = 22.4%) distance. Although not significant, the other two distances also had moderate-to-large proportions of variance accounted for by condition: 50 m (*F*(1, 25) = 2.032, *p* = 0.166, *η*^2^ = 7.5%) and 150 m (*F*(1, 25) = 3.419, *p* = 0.076, *η*^2^ = 12.0%) distances. At each distance, the CTI condition participants’ fixation duration was longer, indicating greater attentional focus than that of the EI condition participants (see Figure 3). The fixation duration was two to three times as long for all three distances among the CTI compared to EI participants: 50 m (*M*_CTI_ = 1.71 s, *M*_EI_ = 0.85 s), 100 m (*M*_CTI_ = 2.11 s, *M*_EI_ = 0.61 s), and 150 m (*M*_CTI_ = 1.75 s, *M*_EI_ = 0.89 s).

### 3.4 Self-efficacy response

**Figure 4:**
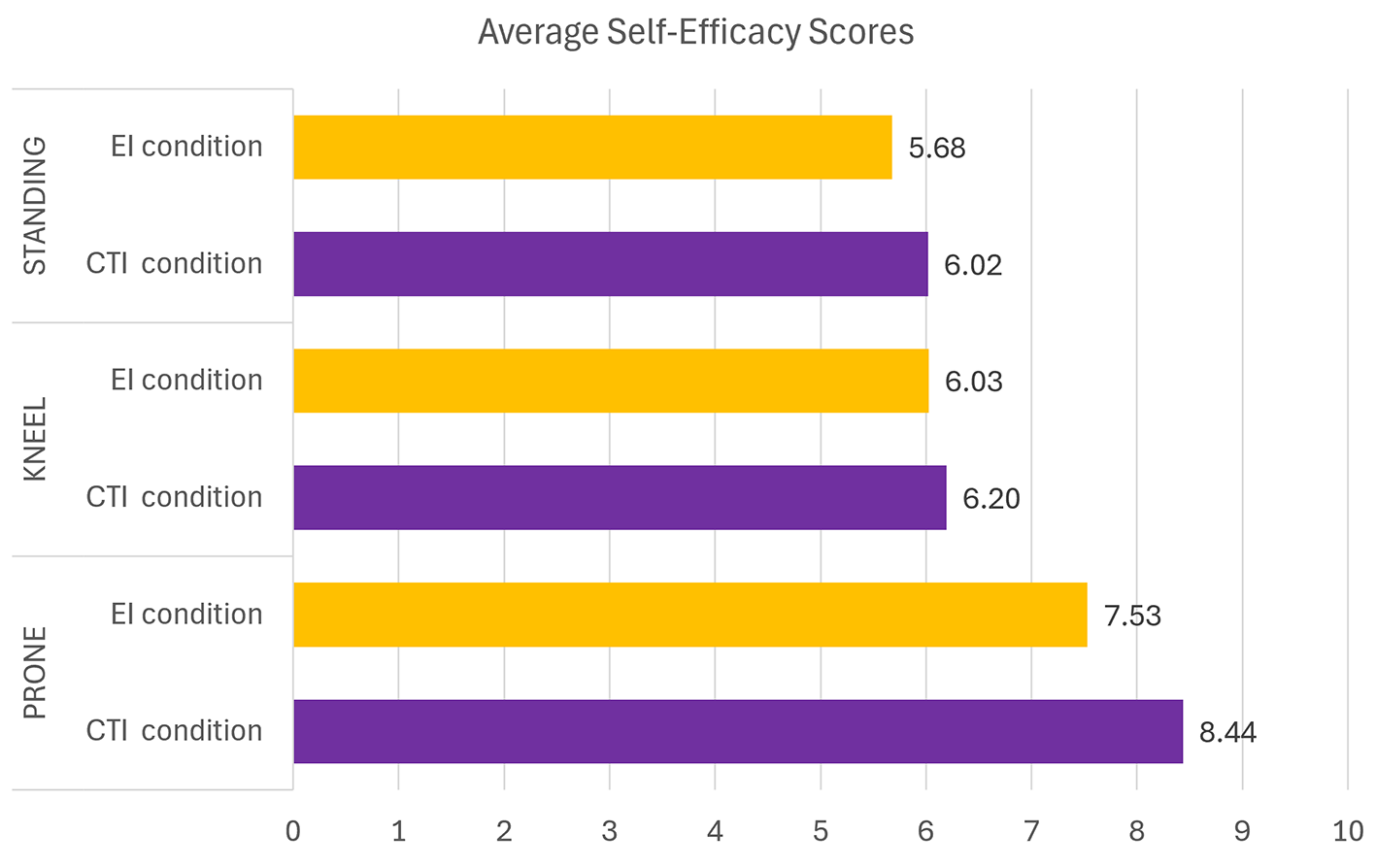
Average self-efficacy values for each position by motivational climate condition.

Box’s test of equality of covariances was not significant (*p* = 0.405), nor was Levene’s test of homogeneity of variances for prone (*p* = 0.361), kneeling (*p* = 0.993), and standing (*p* = 0.522) positions. The self-efficacy values differed significantly by condition, and a moderate proportion of variance was accounted for by condition in the prone position (*F*(1, 58) = 7.800, *p* = 0.007, *η*^2^ = 11.9%). For the other distances, the condition was not significant and accounted for about 1% of their efficacy variance: kneeling (*F*(1, 58) = 0.272, *p* = 0.604, *η*^2^ = 1.0%), and standing (*F*(1, 58) = 0.766, *p* = 0.385, *η*^2^ = 1.3%). In each position, participants in the CTI condition were more efficacious than those in the EI condition (see Figure 4).

### 3.5 Performance response

**Figure 5:**
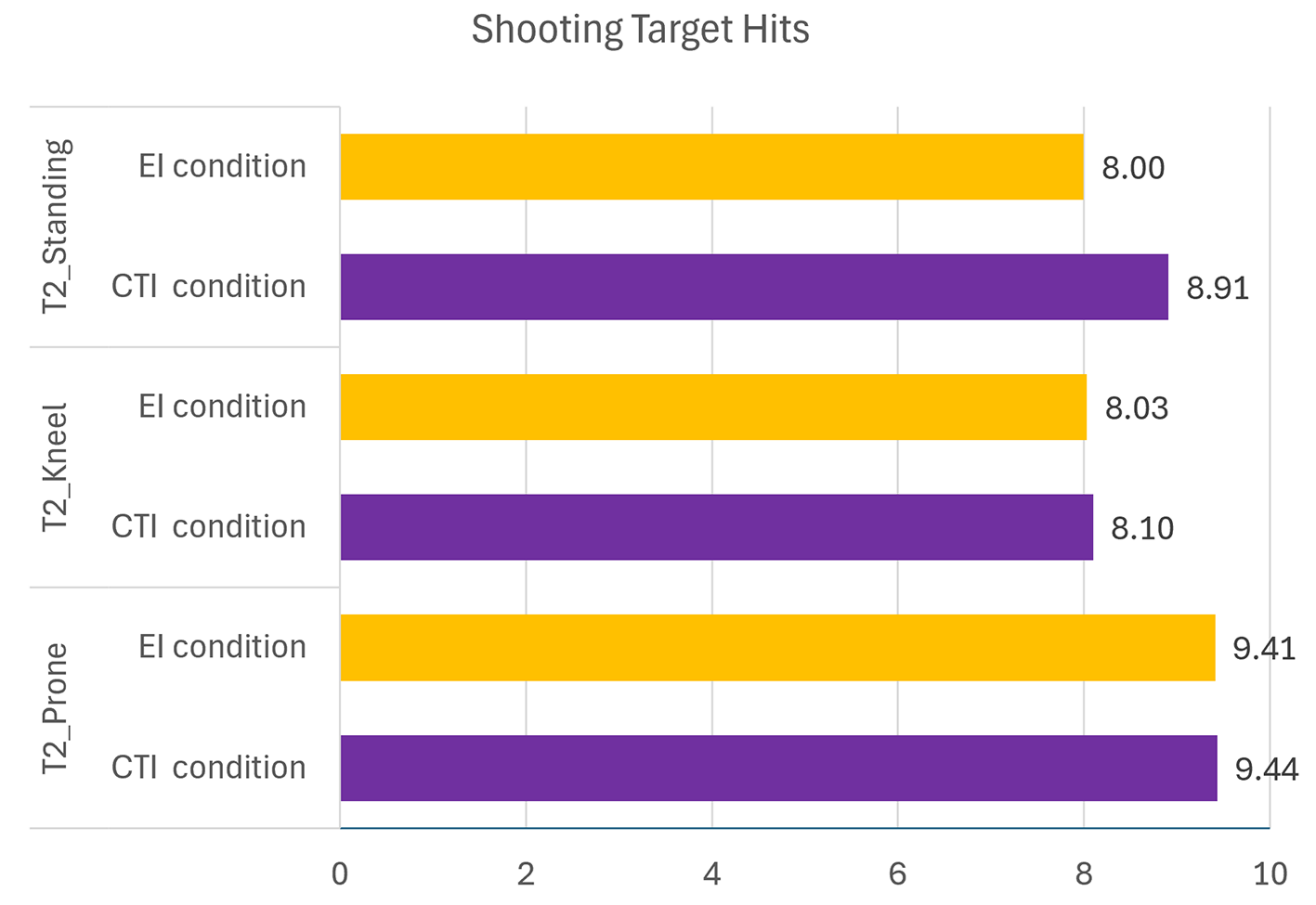
Average targets hit per 15 shots by position and motivational climate condition.

Box’s test of equality of covariances was not significant (*p* = 0.441), nor was Levene’s test of homogeneity of variances for prone (*p* = 0.837), kneeling (*p* = 0.167), and standing (*p* = 0.259) positions. The participants’ number of successful hits did not significantly differ by condition for prone (*F*(1, 59) = 0.001, *p* = 0.980, *η*^2^ = 0%) and kneeling (*F*(1, 59) = 0.005, *p* = 0.945, *η*^2^ = 0%) positions. For the standing position, the difference was nonsignificant, and a small proportion of variance was accounted for by condition (*F*(1, 59) = 0.923, *p* = 0.341, *η*^2^ = 1.5%). Practically, in each position, the CTI condition participants’ marksmanship performance was above that in the EI condition, resulting in an average of over 11% improvement from 23.19 to 26.13 compared to 7.2% improvement from 23.88 to 25.73 (see Figure 5).

## 4 Discussion

This is the first study to examine the effects of CTI and EI experimentally manipulated conditions on self-efficacy, arousal-attentional state, and objectively measured performance (i.e., target hits) with military participants. The novel contribution of the study results provides psychophysiological evidence supporting Nicholls’ proposition that CTI promotes an optimal state for motivation, learning, and performance. Specifically, the CTI participants experienced greater attentional focus, increased self-efficacy, and improved arousal state without entering an anxiety state, which practically resulted in greater relative improvement in performance compared to the EI participants’ performance improvement. These results align with prior research in sport, exercise, and academic (classroom and physical education) contexts. Further, this study extends AGPT experimental research into the military context.

Prior studies involving experimental creation of CTI and EI conditions have consistently supported a CTI climate promotes facilitative interpretations of challenges, such that CTI climate participants perceive their resources outweigh the demands of the activity (e.g., skill training). Hogue and colleagues’ results across multiple studies with young adults have shown with both self-report and physiological markers that people in CTI climates respond with increased effort, appropriate challenge taking, and incorporation of skill cues to improve skill execution without experiencing shame, anxiety, or stress (Hogue et al., 2013; Hogue, 2024; Hogue et al., 2025a; Hogue et al., 2025b). The results of these prior studies indirectly suggest that a sympathetic response is promoted in CTI climate conditions. The current study extends this effort with direct support of a greater sympathetic than vagal response (sympathetic-to-vagal ratio) by CTI participants. Our study indicated that the increased sympathetic response was aligned with improved attentional focus. This supports the CTI feedback, fostering participants’ sympathetic activation, placing them in a more optimal performance and learning state. Importantly, the ROTC cadets did not enter an over-aroused or anxious state, which would have negative consequences for their performance and learning. While further research is needed, this novel result provides the first direct support for the effect of the CTI climate on individuals’ sympathetic arousal response.

Research on measuring attentional focus with eye-tracking has consistently supported that participants’ performance improves when they have an optimal attentional focus (Vickers, 2007). With other marksmanship tasks (i.e., biathlon, basketball free throw shooting), the longer the fixation duration, the more stable individuals’ attentional focus, and the better their shooting performance (Vickers, 1992, 2007). Specifically, research with national team biathletes demonstrated that athletes’ quiet eye (i.e., final fixation duration before/during task execution) was 1.76 s when they hit their targets (Vickers and Williams, 2007). This 1.76 s value is similar to the average fixation duration of the cadets in the CTI condition for all distances when standing and prone, as well as 100 m when kneeling. Prior eye-tracking research has not examined the relationship between fixation duration and motivational climate experiences. However, athletes’ self-reported attentional control survey responses have indicated a positive association with CTI and a negative association with EI characteristics (Domínguez González et al., 2024). Furthermore, the anxiety, shame, and stress psychophysiological responses observed in studies by Smith et al. (2007) plus Hogue and colleagues indicate CTI provides the psychophysiological state to support improved fixation duration (Hogue, 2024; Hogue et al., 2025a; Hogue et al., 2013; Hogue et al., 2025b; Smith et al., 2007). Conversely, these researchers have shown the EI climate fostered an increased anxiety, shame, and stress psychophysiological state which would make it more difficult to attain improved fixation duration and its level of attentional focus (Hogue, 2024; Hogue et al., 2025a; Hogue et al., 2013; Hogue et al., 2025b; Murray and Janelle, 2003; Smith et al., 2007). This is what we saw preliminarily in our study. The CTI participants’ attentional focus increased as represented by their fixation duration, on average, being double that of the EI participants’ eye fixation duration. This study represents the first evidence of this hypothesized relationship, thereby supporting the inclusion of attentional focus through eye-tracking in future motivational climate studies.

Part of AGPT is individuals’ competence or ability perceptions and how they can be influenced by the motivational climate (Nicholls, 1989; Duda and Nicholls, 1992). Research has supported a positive relationship between individuals’ perceived competence or ability and being in a TI climate (Duda and Nicholls, 1992). Measures of individuals’ competence or ability perceptions are more general (e.g., “I am one of the best athletes”, “I am one of the worst athletes”) than self-efficacy (Bandura, 2006). Measuring self-efficacy appraises individuals’ perception of their ability to execute/complete specific skills or tasks (Bandura, 2006). In the academic context, Wood and Bandura (1989) demonstrated that students’ self-efficacy was positively associated with CTI climate characteristics and suppressed in relation to EI climate characteristics. Barkoukis et al. (2010) found a TI intervention, 10 class periods long, increased high school Physical Education students’ self-efficacy. Sport psychology researchers found that athletes’ CTI perceptions were positively associated with their self-efficacy levels (Kavussanu and Roberts, 1996; Zourbanos et al., 2016). Thus, it was expected that receiving CTI climate feedback, rather than EI climate feedback, would result in higher self-efficacy among cadets. This hypothesis was partially supported by cadets’ significantly higher prone self-efficacy, which was the position where the cadets had the most room for efficacy improvement.

Finally, this is the first study based on the AGPT and Caring Climate Framework to objectively assess performance with rifle marksmanship performance. Although there was not a significant difference in performance, there was a meaningful difference during the standing position, which most cadets described as the most difficult due to the lack of rifle support compared to the kneeling and prone positions. It is worth noting that the standing position was also the last position, so cadets could implement what they learned in the prone and kneeling positions while in the standing position. Additionally, the total time spent engaged across the marksmanship activities was 15 min, so this was a brief exposure to the climates. Studies such as Hogue et al. (2013) and Hogue et al. (2025a); exposed participants to researcher-manipulated climate conditions for 20–30 min. It is logical that a longer task session or repeated exposures would produce greater differences. Nonetheless, it is exciting to see evidence that even a single 15-min exposure to the CTI climate may be beneficial for performance.

This study was novel in design and provides support for future research to further examine these relationships; it also had some limitations. Compared to the Hogue et al. (2013) effects of 35% variance due to condition type, the effect sizes of this study were smaller and spanned the small-to-large range (1–22%). Thus, although meaningful effects were observed, often meaningful effects were not found to be significant. This has two primary reasons: (1) the CTI and EI climates were not as distinct in this study as they were in Hogue et al. (2013), and (2) a larger sample size is needed to power the analyses to find this range of small-to-large effects significant. The former reason is related to study design. Hogue et al. (2013) manipulated the climate, with research team members trained for months to produce these distinct climates. In the current study, a “train-the-trainer” design was used by training the senior cadets in how to foster CTI and EI climates. Furthermore (similar to Hogue et al., 2013), leaders were instructed not to create an EI through belittling and disrespectful treatment of their assigned cadets. The manipulation check supported that significantly different climate experiences were developed; however, the EI climate condition was not a strong EI condition. This represents some real-world climates as described by Fischer (2015) when the leaders are also peers; however, it could have been more strongly EI to represent historically common conditions cadets and soldiers experience in boot camp and other contexts. Overall, this explains why the effect sizes were smaller than Hogue et al. (2013). Thus, in the future, researchers using a train-the-trainer model may want to increase the sample size to have power to find the effect sizes seen in this study significant, and/or have longer sessions to increase the time for the climate to develop and influence the participants more.

Relatedly, while the arousal state of the cadets differed in the two conditions, all data was collected early in the morning, so their general initial state was a low arousal, calm, resting state (i.e., vagal or parasympathetic). The marksmanship task, in and of itself, was not responsible for any changes in sympathetic activation, which means it will be a useful task for future studies of arousal responses (i.e., sympathetic activation) in CTI and EI conditions. As marksmanship is a military skill that cadets and soldiers need to regularly train, future researchers could examine the effects of CTI and EI motivational climates over multiple marksmanship training sessions to increase the ecological validity of the future results. Finally, the fixed target for each distance and the variety of strategies used by the cadets, with more or less rapid firing, meant we were not able to tease out quiet eye fixations (i.e., the last fixation before/during task execution) for each shot. Future designs having moving targets (pop-ups) or decision-making sequences will allow for examination of the quiet eye fixations, specifically.

## 5 Conclusion

This study is the first to examine the effects of CTI and EI climate conditions on individuals’ arousal state (i.e., heart rate variability), attentional focus (average fixation duration), self-efficacy, and objective performance during a rifle marksmanship task. The study provided preliminary support for Nicholls’ proposition that a CTI would foster the optimal state for learning and performance, in terms of arousal state and attentional focus (Nicholls, 1989). In particular, the CTI participants’ attentional focus was twice as long as that of the EI participants, providing strong, practical support for the positive effect of experiencing the CTI climate. Furthermore, the CTI cadets were in an active and aroused state, reported greater efficacy, and had improved marksmanship performance. Additional research, building on these initial results, is warranted. Overall, the results of the current study align with existing sport psychology literature on AGPT and the Caring Climate Framework and encourage the continued extension of AGPT and Caring Climate Framework research and application in the military context.

## Data Availability

The datasets presented in this article are not readily available because the UMCIRB consent form did not include permission to share the data collected for this study. Requests to access the datasets should be directed to mooreer22@ecu.edu.
